# Objective Assessment of Foveal Cone Loss Ratio in Surgically Closed Macular Holes Using Adaptive Optics Scanning Laser Ophthalmoscopy

**DOI:** 10.1371/journal.pone.0063786

**Published:** 2013-05-24

**Authors:** Satoshi Yokota, Sotaro Ooto, Masanori Hangai, Kohei Takayama, Naoko Ueda-Arakawa, Yuki Yoshihara, Masaaki Hanebuchi, Nagahisa Yoshimura

**Affiliations:** 1 Department of Ophthalmology and Visual Sciences, Kyoto University Graduate School of Medicine, Kyoto, Japan; 2 NIDEK Co., Ltd, Gamagori, Japan; Case Western Reserve University, United States of America

## Abstract

**Purpose:**

To use adaptive optics scanning laser ophthalmoscopy (AO-SLO) to quantify cone loss ratio in the foveola in order to assess foveal cone status and to investigate relationships between foveal structural abnormalities and visual function in patients with macular hole (MH) after surgery.

**Methods:**

We evaluated 10 normal eyes of 10 healthy volunteers and 19 eyes of 18 patients in whom anatomically successful MH closure had been performed. All subjects underwent a comprehensive ophthalmologic examination that included measurements of spectral-domain optical coherence tomography and AO-SLO.

**Results:**

On AO-SLO regular cone mosaic was seen in all normal eyes whereas dark regions suggesting cone loss were seen in all eyes after MH repair. Visual acuity was better in eyes without dark regions at the center of the fovea than in eyes with them (*P = *0.001). Cone loss ratio in the foveola correlated with postoperative visual acuity (*P*<0.001), mean foveal sensitivity (*P* = 0.029), thinner inner and outer segments at the center of the fovea (*P = *0.002), larger size of the disrupted inner and outer segment junction line (*P = *0.018), and cone outer segment tip line (*P*<0.001). Cone loss ratio in the foveola was significantly greater in eyes that had moderately reflective foveal lesions after surgery (*P = *0.006).

**Conclusions:**

AO-SLO is a useful means of assessing foveal cone damage objectively and quantitatively. The location and extent of cone damage, especially if it involves the foveola, is an important factor determining visual function after MH surgery.

## Introduction

Although macular hole (MH) was first recognized over 100 years ago, there has been renewed interest in the pathophysiology and natural history of MH over the past 2 decades because of an increase in the population of patients susceptible to this condition, as well as recent advances in imaging technologies that have improved our understanding of this disease. [1] This, in turn, has facilitated the development and successful application of improved vitreoretinal surgical techniques. [2] Subsequent refinements in these methods, as well as a better understanding of the predictors for visual outcome, have increased the success of MH surgeries. [3], [4] Despite this, some patients with closed MH have persistent symptoms, including poor visual recovery, central scotoma, or metamorphopsia [5]–[7].

Optical coherence tomography (OCT) has enabled researchers to investigate the microstructural alterations associated with MH; specifically, time-domain OCT (TD-OCT) and higher-resolution spectral-domain OCT (SD-OCT) have been used to visualize structural changes in the photoreceptor layers of eyes with surgically closed MH. [8]–[28] Studies using TD-OCT or SD-OCT have not, however, provided sufficiently clear images of individual photoreceptor cells for identifying specific microstructural abnormalities that might explain persistent visual disturbance in eyes with closed MH.

This failure, which is also associated with other imaging modalities such as scanning laser ophthalmoscopy (SLO), results primarily from aberrations in ocular optics. These can be compensated for by using imaging systems that incorporate adaptive optics (AO) that include a wavefront sensor for measuring aberrations on the ocular surface, and a deformable mirror or spatial light modulator to compensate for aberrations in living eyes. [29]–[33] Adding AO to imaging systems such as flood-illuminated ophthalmoscopes or SLO equipment has allowed researchers to identify abnormalities in individual cone photoreceptors in patients with a variety of retinal diseases [34]–[47].

Recently, we used AO-SLO to investigate the microstructure of eyes with surgically closed MHs. This technique allowed us to see dark areas suggesting cone loss, and to relate this cone damage with macular sensitivity. [47] However, 2 drawbacks of our study included the subjective assessment of cone loss area, and a lack of understanding of how foveola photoreceptor status is related to visual function. Thus, we developed the current follow-up study with 2 main goals: First, to objectively quantify cone loss ratio in the foveola so that we could assess foveal cone status more accurately; and, second, to investigate the relationship between foveal structural abnormalities and visual function in patients with surgically closed MHs.

## Methods

All aspects of our investigation adhered to the tenets of the Declaration of Helsinki, and the study was approved by the institutional review board and the ethics committee at Kyoto University Graduate School of Medicine. The nature of the study and its possible consequences were explained to study candidates, after which written informed consent was obtained from all participants.

### Participants

Subjects for this observational case study were 19 eyes of 18 patients (6 men and 12 women, a mean age of 65.2 years [range: 36–79 years]) with idiopathic MH and 10 normal eyes of 10 healthy subjects (3 men and 7 women, a mean age of 32.3 years). Patients underwent anatomically successful MH closure at the Kyoto University Hospital, Kyoto, Japan, between October 2007 and January 2010; they were then followed for at least 6 months postoperatively. All patients had been diagnosed with stage 2, 3, or 4 idiopathic MH according to the staging system proposed by Gass. [48] Eyes with secondary MH (e.g., caused by trauma, cystoid macular edema, retinal vascular disease, macular pucker, retinal detachment or after laser treatment) were excluded from the study, as were eyes with high myopia (spherical equivalent refractive error of less than -6 diopters or axial length >26.5 mm).

All subjects underwent a comprehensive ophthalmologic examination, which included measurements of best-corrected visual acuity (BCVA) and intraocular pressure, indirect ophthalmoscopy, slit-lamp biomicroscopy with a contact lens, color fundus photography, SD-OCT, and AO-SLO. All parts of the examination were performed during the same visit, which occurred at least 6 months after surgery for patients with MH.

Standard 3-port pars plana vitrectomy (23-gauge system in 15 eyes and 20-gauge system in 4 eyes) for MH repair consisted of a core vitrectomy with intravitreal injection of triamcinolone acetonide (which allowed us to visualize the vitreous gel), surgical creation of a posterior vitreous detachment if it had not yet occurred, internal limiting member (ILM) peeling using 0.05% indocyanine green dye (n = 7) or triamcinolone acetonide (n = 12), and fluid-gas exchange followed by flushing with a mixed nonexpansile concentration of 25% sulfur hexafluoride. Phacoemulsification was performed, and a posterior chamber intraocular lens was implanted prior to vitrectomy in patients with cataract or in those >55 years old. Patients were instructed to remain face down for 7 days after the operation. Anatomic success was defined as the presence of a closed MH on biomicroscopy and SD-OCT.

### AO-SLO System

The AO-SLO system used here was constructed in our laboratory based on previous reports detailing the usefulness of incorporating a wide-field SLO with an AO-SLO. [49], [50] The AO-SLO system is confocal, enabling creation of high-contrast “en face” images in any plane; these images show individual cone photoreceptor cells. The system is composed of 4 primary optical subsystems: the AO subsystem, which includes the wavefront sensor and the spatial light modulator; the high-resolution confocal SLO imaging subsystem; the wide-field imaging subsystem; and the pupil observation subsystem. The wavefront sensor measures aberrations in the whole eye, and the spatial light modulator compensates for these aberrations. The details of the current AO-SLO system are described elsewhere [47], [51].

### AO-SLO Images: Cone Mosaic Features

We obtained AO-SLO images of multiple locations in the macula of each eye by shifting the focus from the retinal nerve fiber layer to the RPE and recording images that showed the cone mosaic. Then, offline, we created a montage of AO-SLO images using Mosaic J (US National Institutes of Health, Bethesda, MD, USA) by selecting the area of interest and selecting each image to be included in the montage from a single frame, without averaging. We confirmed correspondence between each montage and the area of interest by comparing the AO-SLO image with the wide-field images for that eye. The brightness was adjusted to balance the brightness between neighboring images. The center of the fovea, defined here as the center of the foveal avascular zone, was determined from the montage of AO-SLO images by an experienced ophthalmologist (SO) who was blind to all subjects’ visual acuity and other related clinical findings. AO-SLO images were also used to determine the location of the foveola, which was assumed to be a 0.35-mm-diameter area extending from the center of the fovea. To obtain scans with accurate lengths, we corrected the magnification effect in each eye by using the adjusted axial length method reported by Bennett et al. [52].

**Figure 1:**
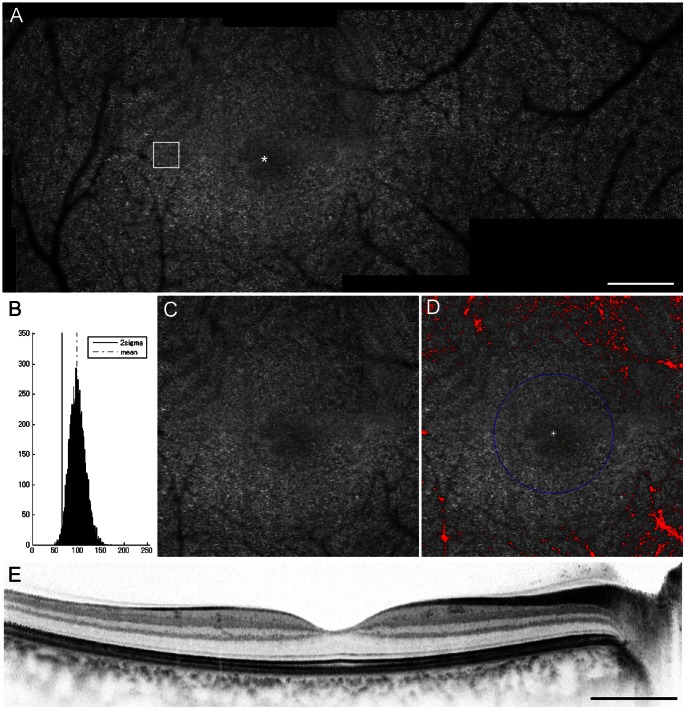
Automated measurement of foveal cone loss ratio. Images of the right eye of a 33-year-old healthy woman with no eye diseases. **A**, A montage of the adaptive optics scanning laser ophthalmoscopy (AO-SLO) images of the area that includes the center of the fovea (asterisk). Scale bar = 200 µm. **B,** Histogram of the reflectivity level of each pixel in the area indicated by white box in A (at 0.5 mm from the center of the fovea). **C**, A high-magnification view that includes the foveola. **D**, Automated detection of cone loss area, defined as the area where the reflectivity level had diminished by 2 standard deviations from the reflectivity level in the unaffected area. The circle indicates the foveola (a 350-µm diameter circle centered on the center of the fovea). Dim pixels with its reflectivity below the threshold are shown in green and red depending on whether they are located inside or outside of the circle, respectively. Cone loss ratio in the foveola was 0.05%.**E**, Spectral-domain OCT (SD-OCT) image. Scale bar = 1000 µm.



Using MATLAB (Mathworks Inc., Natick, MS, USA) and the MATLAB Image Processing Toolbox, we developed an algorithm to automatically measure foveal cone loss ratio (Figure 1). To derive an estimate of cone loss ratio (R) within the circular area around the center of the fovea, the number of dim pixels (Cr) was counted and divided by the total number of pixels in that area (C) using an AO-SLO image that included the center of the fovea:

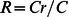
.

For each coordinate (x,y) in the retinal image, Cr was accumulated when the following criteria were satisfied:
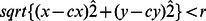


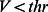
where (*cx, cy)* is the location of the center of the fovea; *r* is the radius of the circular area around the center; *V* is the reflectivity level of the pixel (0–255); and *thr* is the threshold level (0–255; defined as the reflectivity level 2 standard deviations (SD) below levels observed in the unaffected cone mosaic) that determines whether the pixel is dim due to cone loss. Unaffected areas were determined by selecting the area that showed a regular cone mosaic pattern without vascular shadows at 0.5 mm from the center of the fovea.

To assess the inter-observer reproducibility of the cone loss measurements, imaging analysis (by generating an AO-SLO montage image, adjusting the brightness, and choosing the region of interest) was performed by 2 observers (SO and KT) independently on all eyes with closed MH. Images in which each cone was not clearly visible even in the extrafoveal area were excluded from this study.

### SD-OCT: Photoreceptor Layer Features and Retinal Thickness Measurements

SD-OCT was performed using the Spectralis HRA+OCT (Heidelberg Engineering, Dossenheim, Germany). We obtained and evaluated 12 serial radial B-scan images through the fovea of each eye. A freely available image-processing program (ImageJ, US National Institutes of Health, Bethesda, MD, USA) was used to quantify the extent of disruptions of the photoreceptor inner and outer segment junction (IS/OS) line or cone outer segment tip (COST) line. The reflectivity of the IS/OS or COST was measured from within a 40-µm slab, and the IS/OS or COST line was quantified using the plot profile function of the ImageJ software with a 6-pixel fixed-width line. The border of the IS/OS or COST disruption was defined as the line on the grayscale image along which IS/OS or COST reflectivity diminished by 2 SD from the reflectivity of the IS/OS or COST line in the unaffected retina.

**Figure 2:**
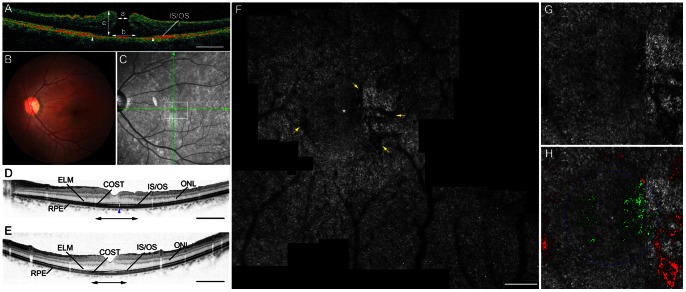
Macular hole case 1. Images of the left eye of a 58-year-old woman with stage 4 idiopathic macular hole (MH) and a Snellen equivalent best-corrected visual acuity (BCVA) of 20/100. **A**, Time-domain optical coherence tomography (OCT) shows a full-thickness MH. The photoreceptor inner and outer segment (IS/OS) junction line is diminished in the area indicated by arrowheads. a: minimum diameter of MH, b: basal diameter of MH, c: MH height. Scale bar = 1000 µm. **B–H**, Images 4 years after surgery, by which point the BCVA had improved to 20/20. **B**, The fundus photography shows a closed MH. **C**, An infrared image with green arrows indicating the directions of the scans producing the images in D and E, and a white box indicating the size of the double-headed arrows in D and E. **D** and **E**, SD-OCT images. **D**, A horizontal-line scan through the center of the fovea, taken in the direction of the horizontal arrow in C, revealing a small disruption of the IS/OS junction (blue arrowhead). ELM: external limiting membrane, ONL: outer nuclear layer, RPE: retinal pigment epithelium. Scale bar = 1000 µm. **E**, A vertical-line scan through the center of the fovea in the direction of the vertical arrow in C, demonstrating that the IS/OS junction is intact. Scale bar = 1000 µm. **F**, AO-SLO images of the area indicated by a white box in C and the double-headed arrows in D and E showing small dark regions (yellow arrows). Dark regions are observed temporal to the fovea corresponding to the area of IS/OS junction disruption in SD-OCT (D). Scale bar = 200 µm.**G**, A high-magnification view that includes the foveola. **H**, Automated detection of cone loss area. The circle indicates the foveola. Dim pixels with its reflectivity below the threshold are shown in green and red depending on whether they are located inside or outside of the circle, respectively. Cone loss ratio in the foveola was 14.07%.

**Figure 3:**
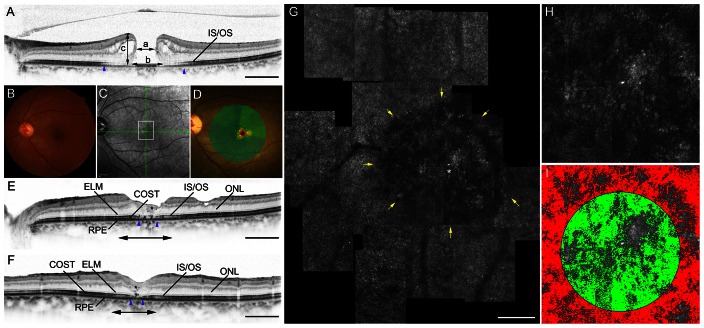
Macular hole case 2. Images of the left eye of a 62-year-old woman with stage 3 idiopathic MH with a Snellen equivalent BCVA of 20/100. **A**, SD-OCT shows a full-thickness MH. The IS/OS junction line is diminished in the area indicated by arrowheads. a: minimum diameter of MH, b: basal diameter of MH, c: MH height. Scale bar = 1000 µm. **B–I**, Images taken 1 year after surgery, by which time BCVA was 20/70. **B**, Fundus photography shows a closed MH. **C**, Infrared image with green arrows indicating the directions of the scans producing the images in E and F, and a white box indicating the size of the double-headed arrows in E and F. **D**, Microperimetry shows central scotoma. **E** and **F**, SD-OCT images. **E**, A horizontal-line scan through the center of the fovea, taken in the direction of the horizontal arrow in C, revealing disruptions of the IS/OS junction (between blue arrowheads). A moderately reflective lesion is seen in the fovea (asterisk). ELM: external limiting membrane, ONL: outer nuclear layer, RPE: retinal pigment epithelium. Scale bar = 1000 µm. **F**, A vertical-line scan through the center of the fovea, in the direction of the vertical arrow in C, demonstrating disruptions of the IS/OS junction (between blue arrowheads). A moderately reflective lesion is seen in the fovea (asterisk). Scale bar = 1000 µm. **G**, AO-SLO images of the area indicated by a white box in C and the double-headed arrows in E and F showing large dark regions (yellow arrows). Dark regions, which are seen all over the fovea, correspond to the area of IS/OS junction disruption on SD-OCT (E and F). Scale bar = 200 µm. **H**, A high-magnification view that includes the foveola. **I**, Automated detection of cone loss area. The circle indicates the foveola. Dim pixels with its reflectivity below the threshold are shown in green and red depending on whether they are located inside or outside of the circle, respectively. Cone loss ratio in the foveola was 68.26%.

The Spectralis HRA+OCT has built-in software that calculates retinal thickness; we used this feature to measure mean central retinal thickness in the 1-mm-diameter area centered on the fixation point in serial B-scan images. B-scans were manually corrected if they were affected by an algorithm failure, such as inaccurately drawn automated boundary lines. In addition, we manually measured anatomic parameters before and after surgery. Using preoperative images, we measured the basal diameter of the MH, MH height (maximal height of elevated foveal retina), and minimum MH diameter using the digital caliper tool built into the OCT system (Figures 2 and 3). Using postoperative SD-OCT images (taken at the same time as the AO-SLO measurement), we also manually measured the thickness of the outer nuclear layer plus Henle’s fiber layer (ONL plus; defined as the distance between the vitreoretinal interface and the external limiting membrane [ELM]), and the thickness of the inner and outer segments (defined as the distance between the ELM and the inner border of the RPE), with all measurements taken at the center of the fovea. The thickness of each retinal layer was the mean thickness determined using vertical and horizontal B-scan images through the fovea.

The quality of each image was evaluated by 2 experienced observers, and only eyes with adequate image quality graded by both observers were included.

### Microperimetry: Retinal Sensitivity Measurements

We used fundus-monitoring microperimetry (MP) to measure retinal sensitivity in 14 eyes at the same visit for AO-SLO measurement. The MP-1 (NIDEK, Padova, Italy) software can be set to automatically track fundus movements and evaluate every acquired frame for shifts in the directions of the X- and Y-axes of the fundus with respect to a reference image obtained by an infrared camera at the beginning of the examination.

We used a 4-2-staircase strategy with a Goldmann size III stimulus against a white background with illumination of 1.27 cd/m^2^ to examine 33 or 41 stimulus locations covering the central 6 or 10 degrees. The differential luminance, defined as the difference between stimulus and background luminances, was 127 cd/m^2^ at 0 decibels (dB) of stimulation. Maximum stimulus attenuation was 20 dB, and the duration of the stimulus was 200 ms.

In the current study, mean foveal sensitivity was defined as the mean of retinal sensitivities measured at 9 different points in the fovea.

### Statistical Analysis

BCVA was measured using the Landolt Chart and expressed as the logarithm of minimal angle of resolution (logMAR). We used paired *t-*tests to compare preoperative factors and postoperative factors. We used Student’s *t*-test to compare BCVA between eyes with dark regions at the center of the fovea and eyes without them. For inter-observer measurements, two-way mixed, average measure intraclass correlation coefficients (ICC [3, K]) were obtained. We calculated the Pearson product moment correlation coefficient to determine associations between cone loss ratio and pre/post-operative variables. We used chi-square tests to correlate numbers of eyes with various factors (sex, stage, ILM staining agent, and structural abnormalities in the fovea on SD-OCT). All statistical evaluations were performed in SPSS v.17 (SPSS Inc., Chicago, IL, USA). Significance was defined as *P*<0.05.

## Results

Patients’ preoperative characteristics are summarized in Table 1. Before surgeries, stage 2 MH was found in 11 eyes, stage 3 in 4 eyes, and stage 4 in 4 eyes. The mean minimum diameter of the MH was 356 µm. The time between the onset of visual symptoms and MH surgery ranged from 2 weeks to 14 months (median: 2 months).

**Table 1:** Baseline characteristics of eyes with macular holes.

Characteristic	Value
Patient age (years), mean ±1 SD (range)	65.2±9.7 (36–79)
Patient gender, no. (%)	
Men	6 (32%)
Women	13 (68%)
Visual acuity, logMAR, mean ±1 SD (range)	0.671±0.253 (0.301–1.097)
Axial length (mm), mean ±1 SD (range)	23.3±0.8 (22.1–25.1)
Symptom duration (months), mean ±1 SD (range)	3.3±3.3 (0.5–14.0)
MH stage, no. (%)	
Stage 2	11 (58%)
Stage 3	4 (21%)
Stage 4	4 (21%)
ILM staining, no. (%)	
with TA	12 (63%)
with ICG	7 (37%)
Minimum diameter of MH (µm), mean ±1 SD (range)	355.7±180.3 (111–630)
Basal diameter of MH (µm), mean ±1 SD (range)	720.5±338.5 (152–1333)
MH height (µm), mean ±1 SD (range)	448.2±53.0 (370–507)
IS/OS decreased reflectivity size (µm), mean ±1 SD (range)	1607.3±893.9 (263–4156)

SD: standard deviation, log MAR: logarithm of minimal angle of resolution, MH: macular hole, ILM: internal limiting membrane, TA: triamcinolone acetonide, ICG: indocyanine green, IS/OS: junction of the photoreceptor inner and outer segment layers.

Postoperative characteristics of the eyes (at the time of AO-SLO imaging) are summarized in Table 2. The time between surgery and measurement ranged from 6 months to 4 years (median: 13 months). Surgery significantly improved mean logMAR from 0.671 to 0.214 (*P*<0.001, paired *t*-test). Mean IS/OS decreased reflectivity size was reduced from 1607 µm to 310 µm (*P*<0.001, paired *t*-test).

**Table 2:** Postoperative characteristics of macular hole patients.

Characteristic	Value
Postoperative time (months), mean ±1 SD (range)	20.4±16.1 (6–49)
Postoperative visual acuity, logMAR, mean ±1 SD (range)	0.214±0.279 (−0.079–1.000)
Mean foveal sensitivity (dB), mean ±1 SD (range)	15.18±3.02 (9.3–20.0)
Thickness of ONL plus at the foveal center (µm), mean ±1 SD (range)	101.7±31.1 (62–195)
Thickness of inner and outer segments at the foveal center (µm), mean ±1 SD (range)	71.5±24.5 (22–113)
Mean central retinal thickness (µm), mean ±1 SD (range)	290.4±33.7 (225–350)
IS/OS decreased reflectivity size (µm), mean ±1 SD (range)	310.4±472.1 (0–1860)
COST decreased reflectivity size (µm), mean ±1 SD (range)	192.7±200.5 (0–587)

SD: standard deviation, logMAR: logarithm of minimal angle of resolution, ONL plus: outer nuclear layer plus Henle’s fiber layer, IS: inner segment, OS: outer segment, IS/OS: photoreceptor inner and outer segment, COST: cone outer segment tip.

The mean central retinal thickness (1-mm center) was 290±33 µm in the eyes with MH after surgery, which was compared to that in normal eyes (279±20 µm, *P* = 0.335). The thicknesses of the ONL plus and the inner and outer segments at the center of the fovea were 102±31 µm and 72±25 µm, respectively, in eyes with a surgically closed MH, and 104±13 µm and 85±5 µm in normal eyes (*P = *0.804 and 0.026, respectively, *t*-test).

**Figure 4:**
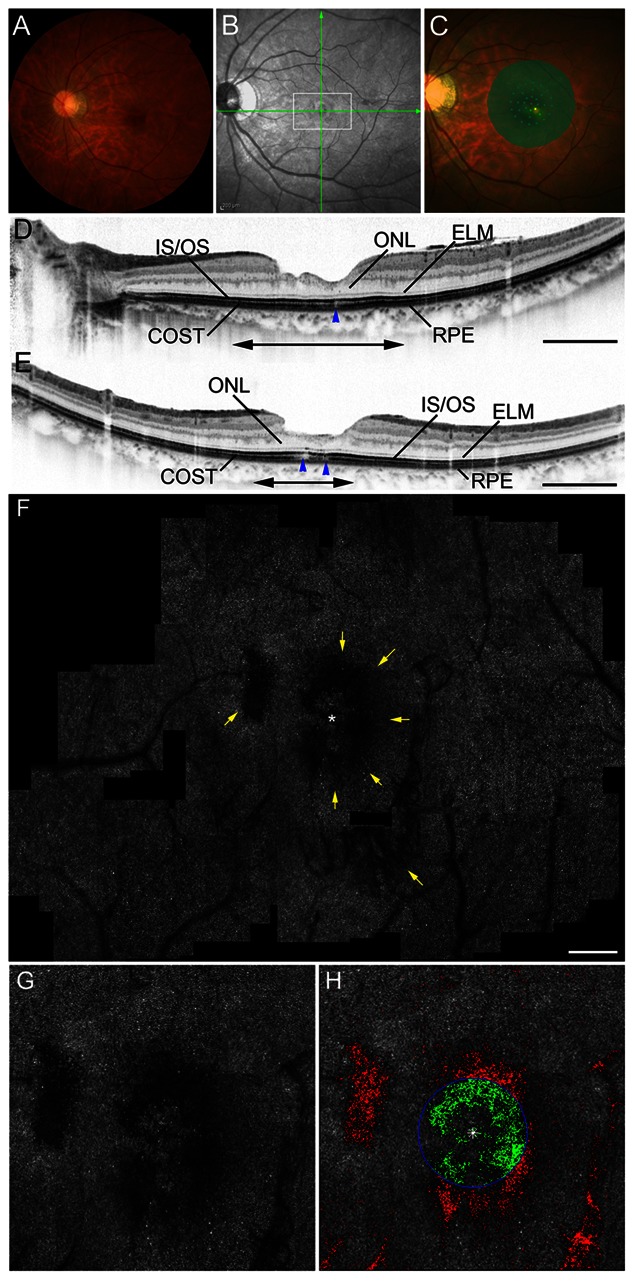
Macular hole case 3. Images of the left eye of a 36-year-old woman with surgically closed MH with a Snellen equivalent BCVA of 20/20 (improved from a presurgical BCVA of 20/70 as a result of stage 4 MH). **A,** Fundus photography shows a closed MH. **B,** An infrared image with green arrows indicating the directions of the scans producing the images in D and E, and a white box indicating the extent of the double-headed arrows in D and E. **C**, Microperimetry shows relative scotoma. **D** and **E,** SD-OCT images. **D,** A horizontal-line scan through the center of the fovea, taken in the direction of the horizontal arrow in B, revealing that the line representing the IS/OS junction is intact, whereas the line representing the cone outer segment tip (COST) is disrupted (blue arrowhead). ELM: external limiting membrane, ONL: outer nuclear layer, RPE: retinal pigment epithelium. Scale bar = 1000 µm. **E,** A vertical-line scan through the center of the fovea in the direction of the vertical arrow in B, demonstrating that the IS/OS junction is almost continuous, whereas COST is disrupted (blue arrowheads). Scale bar = 1000 µm. **F,** AO-SLO images of the area indicated by the white box in B and the double-headed arrows in D and E showing irregular dark regions (yellow arrows). Note that dark regions are seen beside and within the foveola but are not seen in the center of the fovea. Dark regions correspond to the area of COST disruption on SD-OCT (D and E). Scale bar = 200 µm. **G**, A high-magnification view that includes the foveola. **H**, Automated detection of cone loss area. The circle indicates the foveola. Dim pixels with its reflectivity below the threshold are shown in green and red depending on whether they are located inside or outside of the circle, respectively. Cone loss ratio in the foveola was 48.62%.

The AO-SLO revealed dark regions that were seen in all eyes after MH repair. Dark regions on the AO-SLO were confined to the pre-operative MH area. The dark regions corresponded to areas of IS/OS junction disruption (Figures 2 and 3) or to areas where the IS/OS line was almost intact but the COST line was disrupted (Figure 4) as seen on post-operative SD-OCT.

Where dark regions were seen at the center of the fovea (n = 8 eyes; Figure 3), mean logMAR visual acuity was significantly lower (0.434±0.029) than where dark regions avoided the center of the fovea (n = 11 eyes; Figures 2 and 4; mean logMAR visual acuity = 0.054±0.120; *P = *0.001, *t-*test).

**Figure 5:**
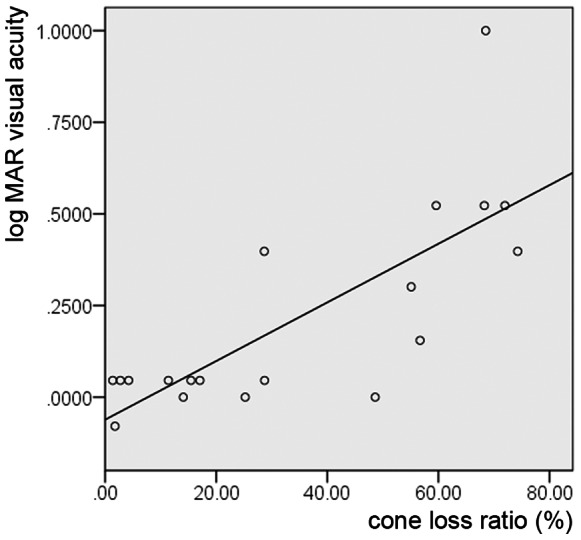
Cone loss ratio and visual acuity. Relationship between cone loss ratio in the foveola with best-corrected visual acuity in eyes with surgically closed MH. Cone loss ratio in the foveola was significantly correlated with the logarithm of the minimum angle of resolution (logMAR) (*P*<0.001, r = 0.767).

In normal eyes, cone loss ratio in the foveola was 0.11±0.06%. In contrast, cone loss ratio of the foveola ranged from 1.77% to 74.28% in eyes with surgically closed MH. Inter-observer ICC was 0.912 for calculating cone loss ratio in eyes with closed MH. Cone loss ratio did not correlate with preoperative or intraoperative factors (age, gender, visual acuity, axial length, symptom duration, MH stage, minimal diameter of MH, basal diameter of MH, MH height, IS/OS decreased size, and ILM staining agent). However, cone loss ratio in the foveola correlated with postoperative log MAR visual acuity (*P*<0.001, r = 0.767) (Figure 5) and mean foveal sensitivity (*P = *0.029, r = −0.545) (Table 3). Further, the cone loss ratio in the foveola was correlated with postoperative thinner inner and outer segments at the center of the fovea (*P = *0.002, r = −0.684), larger IS/OS decreased reflectivity size (*P = *0.018, r = 0.536), and larger COST decreased reflectivity size (*P*<0.001, r = 0.840) (Table 3). On AO-SLO images, cone loss ratio in the foveola was significantly greater in eyes that had moderately reflective foveal lesions (Figure 3) after surgery (*P = *0.006) (Table 4).

**Table 3:** Correlation between cone loss ratio in the foveola and both pre- and postoperative characteristics of eyes with macular holes.

Characteristic	Cone loss ratio	
	*r*	*P*
**Preoperative**		
Patient age	0.092	0.709
Visual acuity, logMAR	0.391	0.098
Axial length	0.185	0.493
Symptom duration	0.435	0.063
Minimum diameter of MH	0.340	0.197
Basal diameter of MH	0.450	0.092
MH height	0.011	0.968
IS/OS decreased reflectivity size	0.330	0.211
**Postoperative**		
Visual acuity, logMAR	0.767	**<0.001**
Mean foveal sensitivity	−0.545	**0.029**
Central retinal thickness	−0.314	0.205
Thickness of ONL at the foveal center	0.045	0.858
Thickness of inner and outer segments at the foveal center	−0.684	**0.002**
IS/OS decreased reflectivity size	0.536	**0.018**
COST decreased reflectivity size	0.840	**<0.001**

log MAR: logarithm of minimal angle of resolution, MH: macular hole, IS/OS: photoreceptor inner and outer segment junction, ONL plus: outer nuclear layer plus Henle’s fiber layer, COST: cone outer segment tip.

**Table 4:** Relationships between cone loss ratio in the foveola and pre-, intra-, and postoperative characteristics of eyes with macular hole.

Characteristic		Cone loss ratio (%)	*P* *
**Preoperative**			
Gender	Men	22.2±26.1	0.183
	Women	40.0±26.1	
MH stage	2	28.2±25.1	0.245
	3, 4	43.0±.28.3	
**Intraoperative**			
ILM staining agent	TA	31.1±26.2	0.495
	ICG	40.1±28.9	
**Postoperative**			
Moderately reflective foveal lesion	−	23.6±19.4	**0.006**
	+	57.8±26.7	

* As determined by a Student’s *t*-test.

MH: macular hole, ILM: internal limiting membrane, TA: triamcinolone acetonide, ICG: indocyanine green.

Cone loss ratio in the foveola limited to eyes more than 1 year after surgery for MH closure (n = 14) correlated with postoperative logMAR visual acuity (*P = *0.001, r = 0.767) and mean foveal sensitivity (*P* = 0.046, r = −0.611).

Cone loss ratio in the 0.5-mm-diameter area extending from the center of the fovea also correlated with postoperative logMAR visual acuity (*P = *0.003, r = 0.643) and mean foveal sensitivity (*P = *0.049, r = −0.500).

## Discussion

Using conventional TD-OCT, [8]–[18] ultrahigh-resolution OCT, [19], [20] and SD-OCT, [21]–[28] several previous studies have found a possible association between the integrity of the photoreceptor layer and visual improvement after successful MH repair. Many investigators have observed a disrupted photoreceptor IS/OS junction boundary and a disrupted ELM that could be associated with postoperative visual impairment. [10], [11], [13], [14], [19], [20], [22]–[25] Using AO-SLO, we previously showed cone loss in eyes with surgically closed MH and a correlation between this anatomic finding and retinal sensitivity. [47] However, because our AO-SLO system cannot always resolve individual cone photoreceptors near the foveal center, it has been difficult to evaluate the cone status of the foveola. In the current study, use of a new program focused on the reflectivity of the cone mosaic allowed us to objectively assess cone loss area in the foveola in eyes with surgically closed MH. Our current work has provided the evidence that cone damage in the foveola well reflects visual disturbance after MH surgery.

Dark regions representing cone loss were seen in all eyes with surgically closed MH, which is consistent with our previous study. [47] However, cone loss areas were sometimes multiple and irregularly shaped (Figures 2–4); this suggests that manual segmentation of the cone loss area may be somewhat subjective and inaccurate. In the present study, we objectively quantified the cone loss ratio in the foveola to assess the foveal cone status more accurately. We automatically analyzed cone loss area in each eye and applied a program to analyze the cone loss ratio in the foveola. Because the program’s algorithm depends on the reflectivity of the cones, cone loss ratio could be measured even in eyes where the outlines of individual cones in the foveola were not clearly visible. The inter-observer reproducibility was high, and cone loss ratio in normal eyes was almost zero, indicating that this method using the AO-SLO system permits reliable measurement of cone loss ratio in the foveola. This method can be used to investigate photoreceptor structural abnormalities in other diseases, such as inherited retinal degeneration, central serous chorioretinopathy, idiopathic macular telangiectasia, and macular microhole, in which cone loss can be observed using an AO fundus camera or AO-SLO [35]–[47].

Because it is confocal, the AO-SLO system allows us to create high-contrast en face images. The axial resolution of OCT is higher than that of AO-SLO; however, it is difficult to interpret C-scan OCT images because the spherical shape of the eyeball prevents these images from showing a single layer of the retina. Moreover, commercially available SD-OCT systems (without AO) have a lateral resolution of approximately 20 µm, while the AO-SLO - which has a lateral resolution of 3 µm - can detect abnormalities that are less than the 20 µm lateral resolution of OCT. Thus, use of the AO-SLO system provides more precise evaluations of the 2-dimensional extent of photoreceptor alterations than does commercially available SD-OCT. In the current study, worse visual acuity was found on AO-SLO images in eyes with cone loss in the center of the fovea, suggesting that functional impairment is closely related to photoreceptor alterations in the center of the fovea. Good BCVA was observed in eyes with dark regions proximal to the foveola but not in the center of the fovea (Figure 4). In addition, cone loss ratio in the foveola was highly correlated with visual acuity and mean foveal sensitivity. Thus, there appear to be several important factors for determining postoperative visual function, including the location and extent of cone damage and, especially, how the foveola is involved.

The high reflectance of the cone mosaic in AO-SLO is thought to be caused by reflectance from both the IS/OS and the COST in the normal retina. [53] In fact, the current study showed that cone loss ratio of the foveola was correlated with larger IS/OS decreased reflectivity size and larger COST decreased reflectivity size. However, dark areas were also seen on AO-SLO images in the areas where, on SD-OCT, the IS/OS line was almost intact but the COST line was disrupted (Figure 4). This finding is consistent with the results of a study of eyes with macular microholes, in which the dark area seen on AO fundus camera images corresponded with the areas where the COST line, rather than the IS/OS, was disrupted on SD-OCT images. [39] Thus, the COST line probably plays a more important role in the reflectance of the photoreceptor mosaic on AO-imaging devices.

Another significant finding of our study was that cone loss ratio in the foveola on AO-SLO correlated with thinner inner and outer segments on SD-OCT after surgical closure of MH. We also observed that a moderately reflective lesion on SD-OCT, considered to represent glial cell proliferative events, [20], [24] was associated with large dark regions within the foveola on AO-SLO. Thus, decreased inner and outer segment thickness and a moderately reflective lesion on SD-OCT may reflect severe structural disturbance of the photoreceptor layer.

The dynamic healing process of MHs after surgical repair has been analyzed using SD-OCT. [23]–[26] Recently, Bottoni et al. reported that the ELM is the first structure to recover after MH closure, and foveal cysts may develop during follow-up, and in the presence of an intact ONL, they may gradually fill with complete recovery of the IS/OS. [26] Further studies using AO-SLO in combination with SD-OCT to analyze the progressive photoreceptor changes for more than 1 year will reveal the detailed dynamic structural changes of the photoreceptors after MH repair.

A limitation of this study is (1) its retrospective nature and the interval to postoperative AO-SLO imaging was therefore not necessarily the same for each patient. The defect area is likely to decrease for several months after surgery and may account for sustained improvements in visual acuity. [26] To minimize the effects of variations in this interval, we performed AO-SLO measurements at least 6 months after surgery and used the BCVA measured at the time of AO-SLO examination for analysis. In addition, subclass-analysis using eyes that had undergone MH surgery more than 1 year previously showed similar results. (2) We could not evaluate AO-SLO images preoperatively, because we were unable to adequately focus on photoreceptors in one plane (due to the roll-up of photoreceptors) with the AO-SLO system. (3) Dark areas outside the foveola can influence the patient’s visual acuity. In the current study, we calculated cone loss ratio in the 0.5-mm-diameter area extending from the center of the fovea and determined correlation with postoperative logMAR visual acuity and mean foveal sensitivity. (4) This method would provide false positives for cone loss in regions of retinal vasculature. However, in the current study, cone loss ratio was measured within the foveal avascular zone.

In conclusion, our study shows that AO-SLO could be used to detect foveal photoreceptor damage objectively and quantitatively; further, we also determined that cone loss ratio of the foveola automatically detected using this method is closely associated with visual acuity in eyes with closed MH. In the future, we hope to perform prospective longitudinal studies to learn more about the effects of cone loss areas on the healing process of MH, with the hope that this knowledge will point the way to better management of this disease.
